# Supplementary material for: Organic light emitting board for dynamic interactive display
Source: Nat Commun. 2017 Apr 13;8:14964. doi: 10.1038/ncomms14964 (PMC5399280; doi:10.1038/ncomms14964)
Supplement: Supplementary Information — Supplementary Figures, Supplementary Tables and Supplementary Notes [file ncomms14964-s1.pdf]

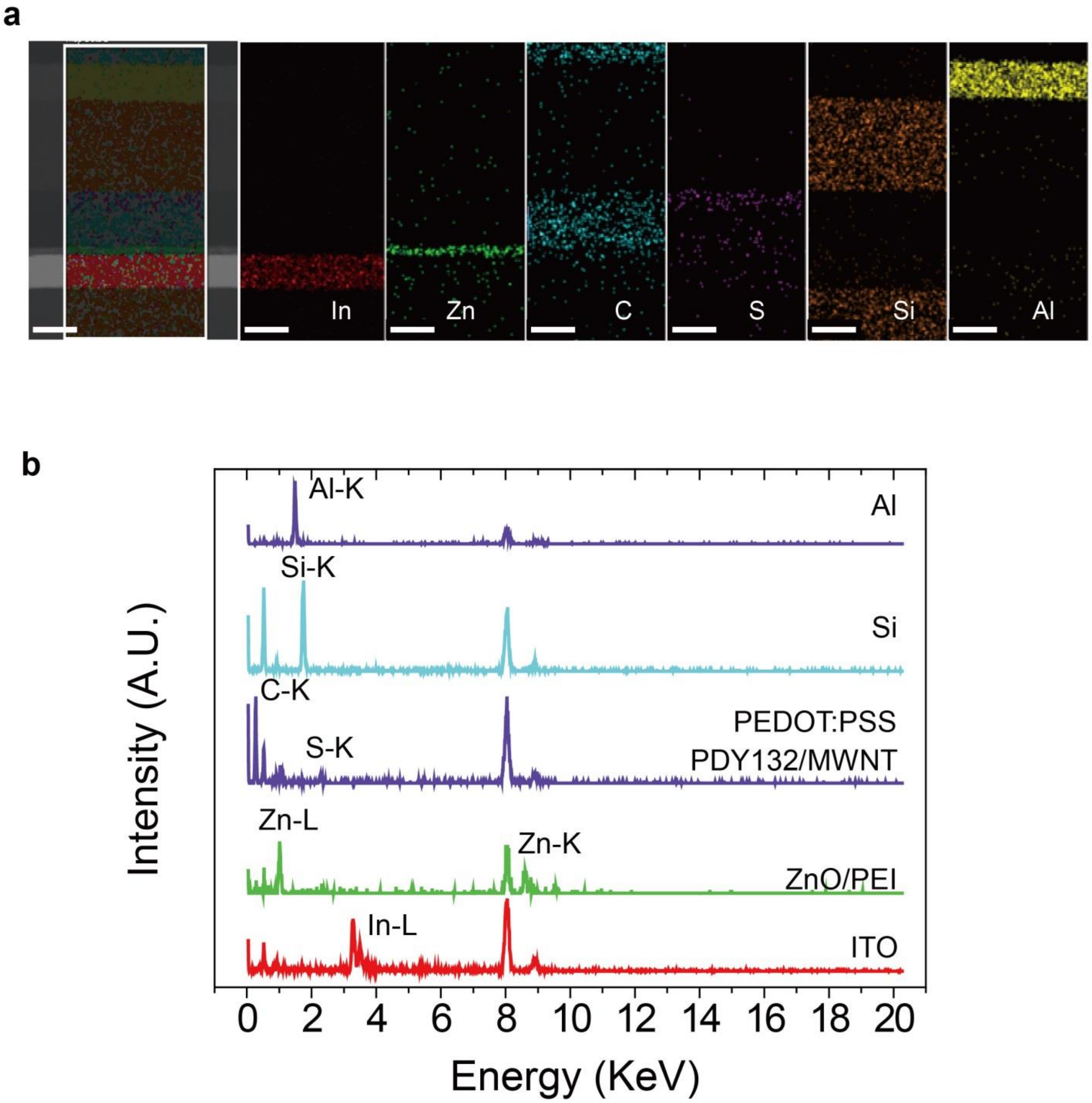

Supplementary Figure 1. Chemical analysis of the constituent layers of a parallel AC EL device (**a**) 2 dimensional mapping and (**b**) energy spectra of TEM-EDX measurements with multivariate statistical analysis of characteristic atomic elements of the constituent layers. The scale bar is 100 nm.

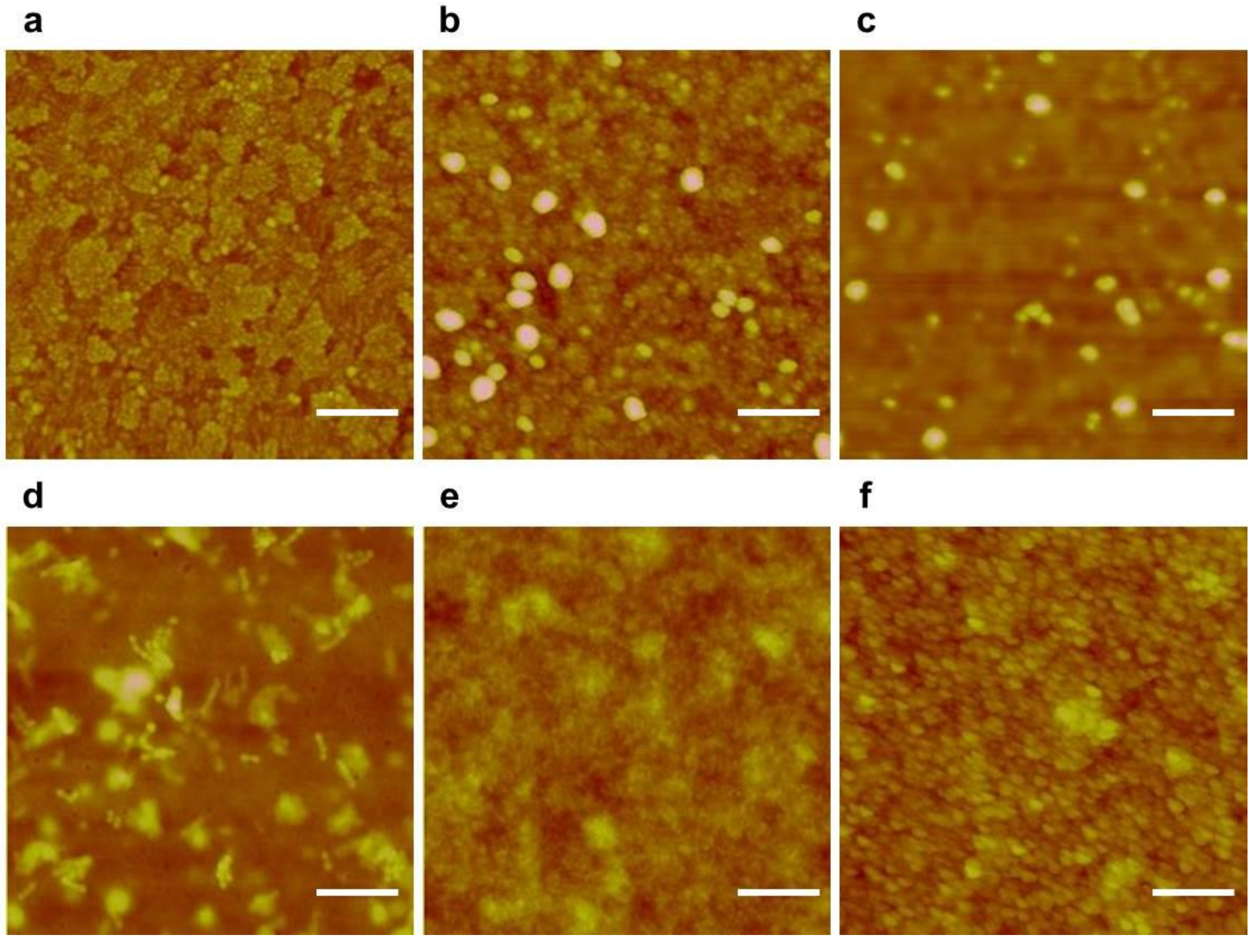

Supplementary Figure 2. TM-AFM images in height contrast of the surface morphology of the constituent layers of a parallel AC EL device. The images indicate very low RMS roughness of (**a**) ITO (2.291 nm), (**b**) ZnO (4.849 nm), (**c**) PEI (3.258 nm), (**d**) Super Yellow/MWNT (3.209 nm), (**e**) PEDOT:PSS (2.378 nm), and (**f**) SiO_2 (3.741 nm). The scale bar is 500 nm.

Supplementary Note 1:

Operation mechanism of an OLEB

Our device can be regarded equivalently in device architecture as vertically stacked with a mirror plane at the centre of a floating Al electrode with top and bottom ITO electrodes (Supplementary Fig. 3). For convenience, we assume that the bottom and top ITO electrodes correspond to left and right ITO ones, respectively. Half of the voltage bias applied at the two ITO electrodes is developed between the bottom ITO and floating Al electrode. For instance, half of the voltage drop occurs at a top Al electrode when positive voltage is imposed on the left ITO electrode (See a red box of Supplementary Fig. 3a). In this situation, light is emitted from the overlapped area between the ITO in the left side and Al electrode. To facilitate field driven hole injection, we also introduced a PEDOT:PSS carrier injection layer. Upon negative bias at the ITO in the left side, holes in the PEDOT:PSS layer are injected to an emitting layer and excitons are subsequently formed with the electrons from ITO electrode, giving rise to light emission upon recombination (Supplementary Fig. 3b). However, no light was emitted from the overlapped Al and ITO on the right side, owing to high injection barriers for holes and electrons at the Al and ITO electrodes, respectively. When the field is switched to a positive bias at the ITO in the left, the overlapped Al and ITO in the right side emits light while no emission occurs at the overlapped area of Al and ITO in the left side (Supplementary Fig. 3c and 3d).

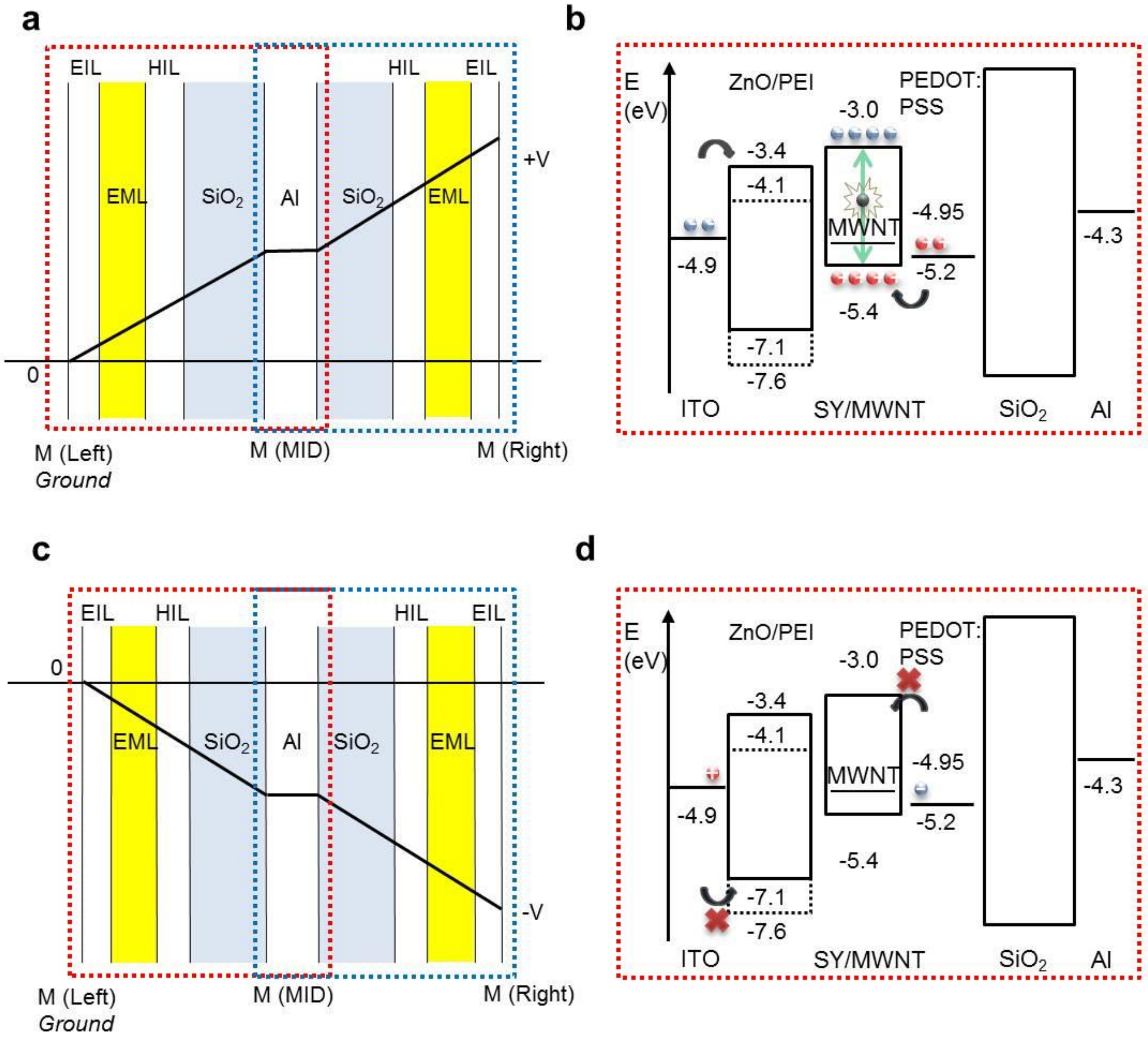

Supplementary Figure 3. Operation mechanism of a parallel AC EL device based on a device model equivalent with that of a parallel AC EL device. (**a**) Voltage potential diagram of device structure consisting of ITO/ZnO/PEI/EML/SiO_2/Al/SiO_2/EML/PEI/ZnO/ITO which is equivalent in device architecture with that of a parallel AC EL device. A positive electric field is assumed to be exerted on the right ITO electrode. (**b**) The energy levels for operation mechanism of the parallel AC EL device. The energy diagram corresponds to that of the LEU1 of a. A positive electric field is again assumed to be exerted on the right ITO electrode. (**c**) Voltage potential diagram of device structure equivalent in device architecture to a parallel AC EL device. A negative electric field is assumed to be exerted on the right ITO electrode. (**d**) The energy levels for operation mechanism of the parallel AC EL device. The energy diagram corresponds to that of the LEU2 of Figure 1b. A negative electric field is again assumed to be exerted on the right ITO electrode.

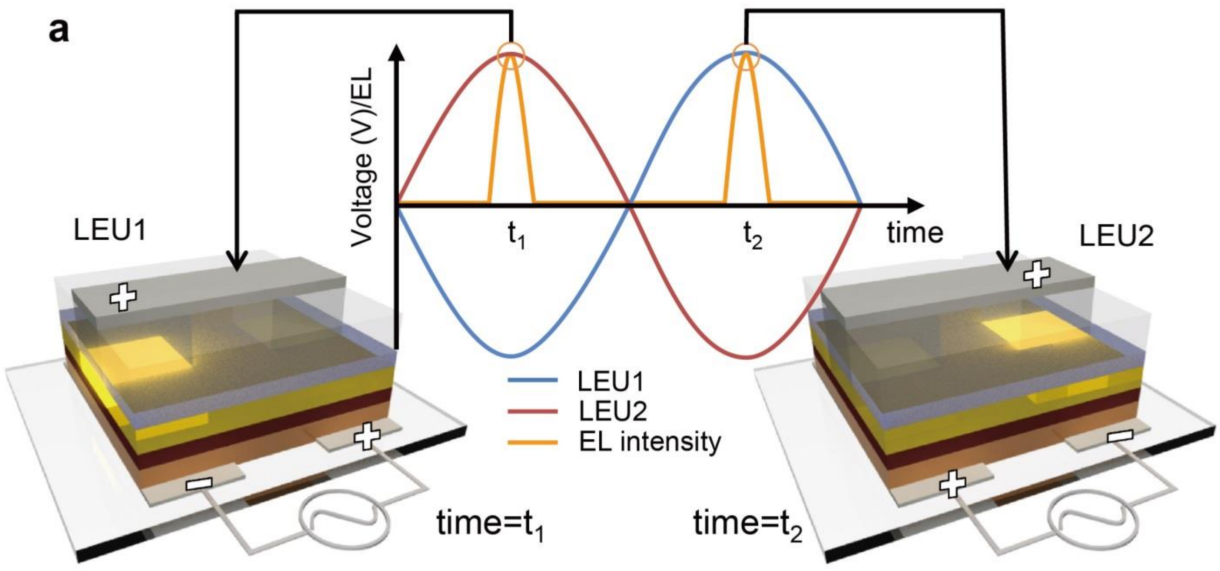

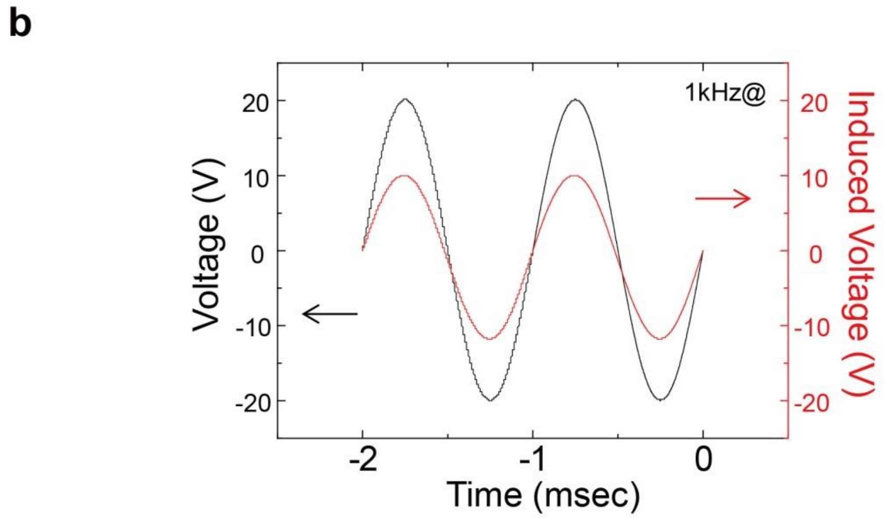

Supplementary Figure 4. Light emission principle of a parallel AC EL device under AC field. (**a**) Schematic of operation mechanism of a parallel AC EL device with two LEUs under AC bias. (**b**) The time-resolved induced voltage signals of top electrode of a parallel AC EL device under AC voltage with a frequency of 1 kHz.

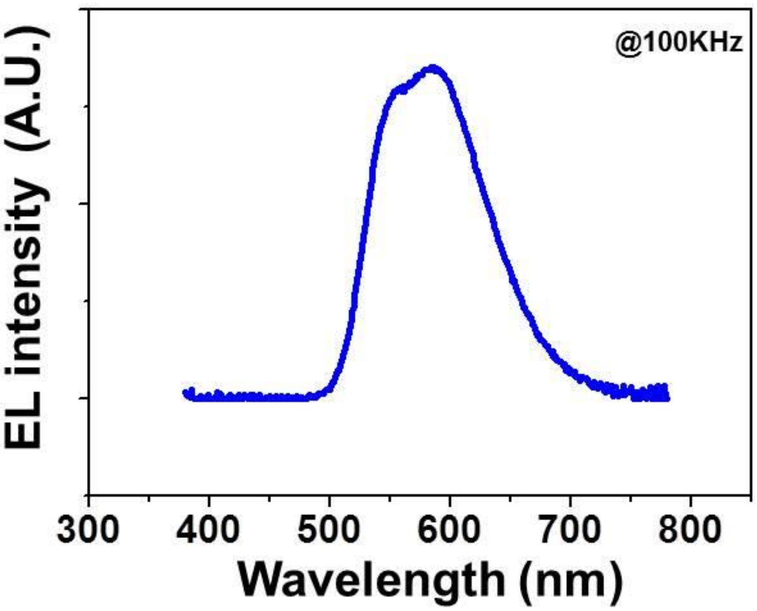

Supplementary Figure 5. An EL spectrum of a parallel AC EL device with a frequency of 100 kHz.

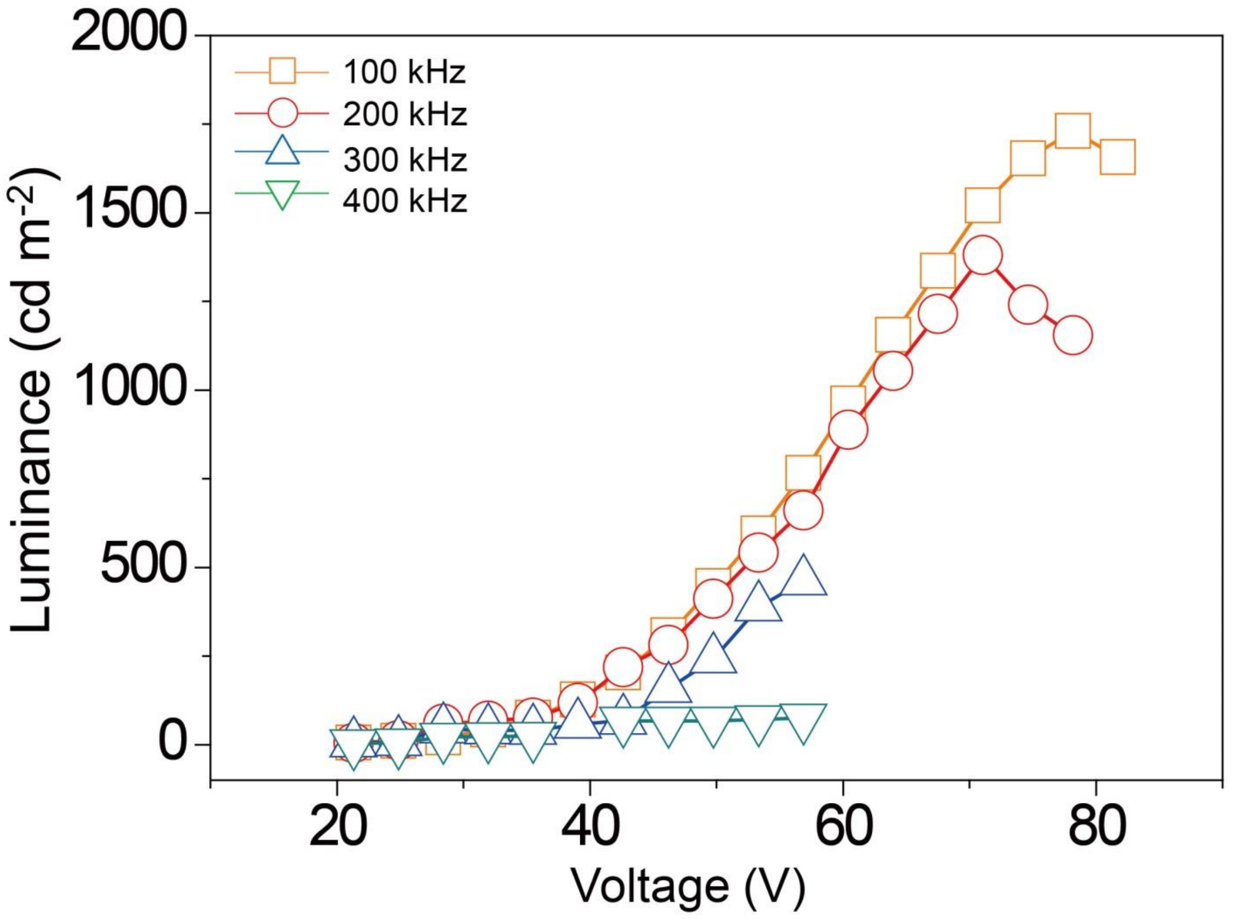

Supplementary Figure 6. Frequency dependent operation of a parallel AC EL device. Luminance versus voltage (L–V) characteristics of a parallel AC EL device operated at various frequencies.

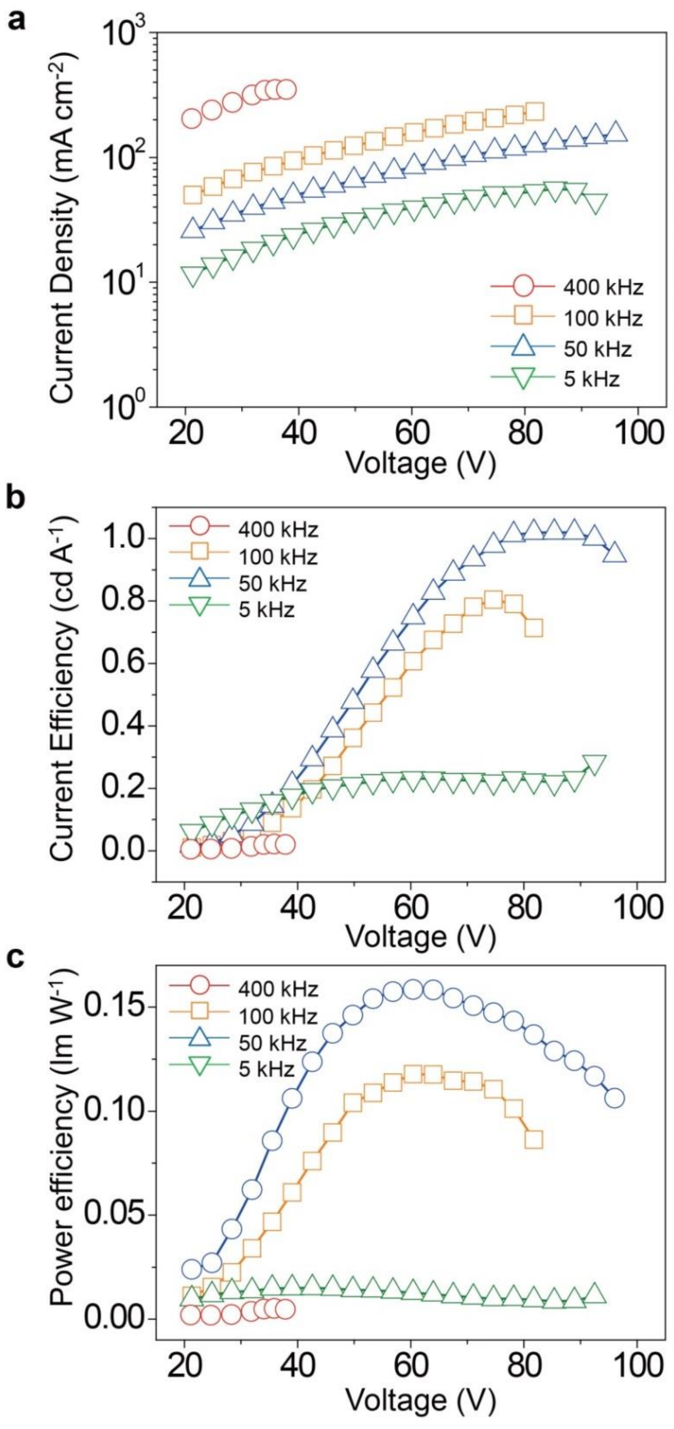

Supplementary Figure 7. Device performance of a parallel AC EL device. Characteristics with respect to voltage (V) of a parallel AC EL device. (**a**) Current density (mA cm^{-2}) (**b**), Current efficiency (cd A^{-1}) and (**c**) power efficiency (lm W^{-1}) characteristics with respect to voltage (V) of a LEU of a parallel AC EL device examined at AC frequencies of 5, 50, 100, and 400 kHz.

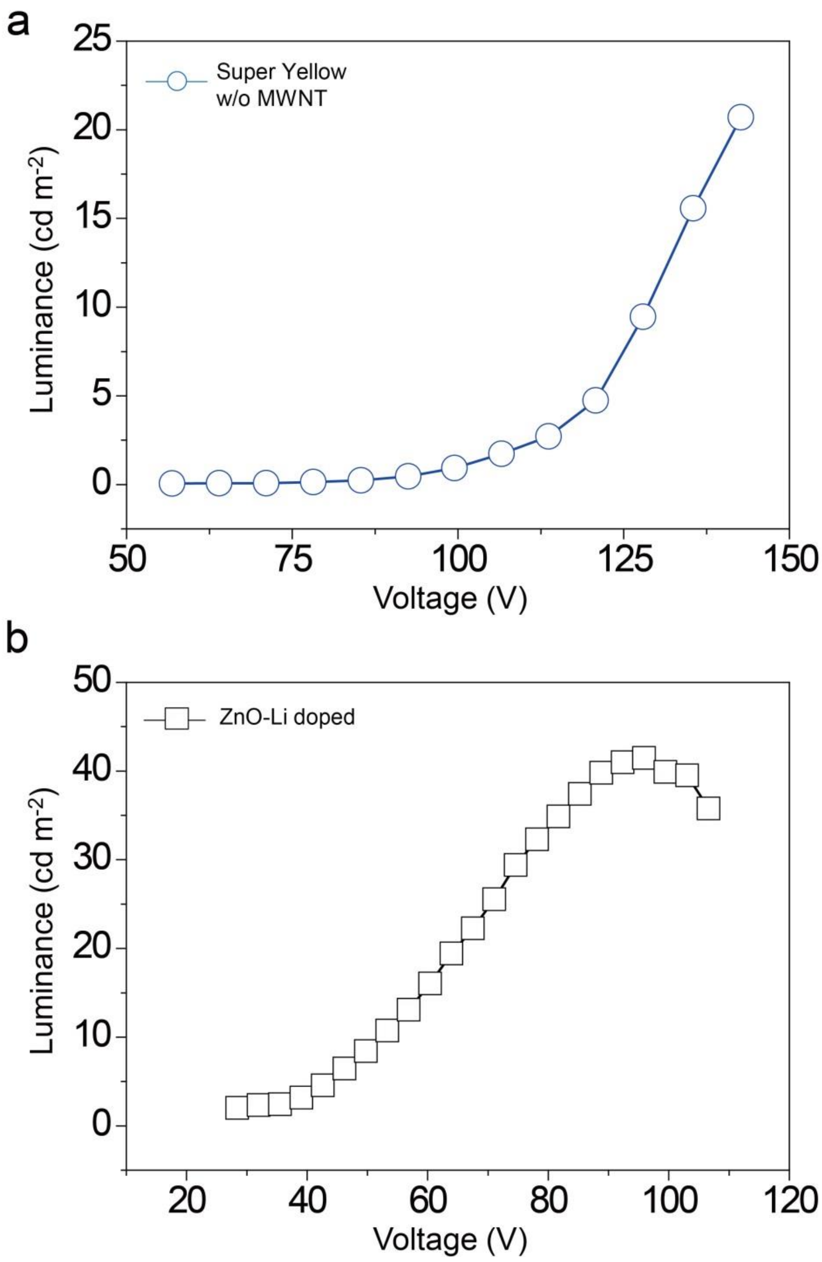

Supplementary Figure 8. Effects of SWNTs as well as Li-doping in ZnO on the performance of a parallel AC EL device. Luminance versus voltage (L–V) characteristics of (a) a parallel AC EL device without SWNTs and (b) a parallel AC EL device with a ZnO-Li doped electron transport layer.

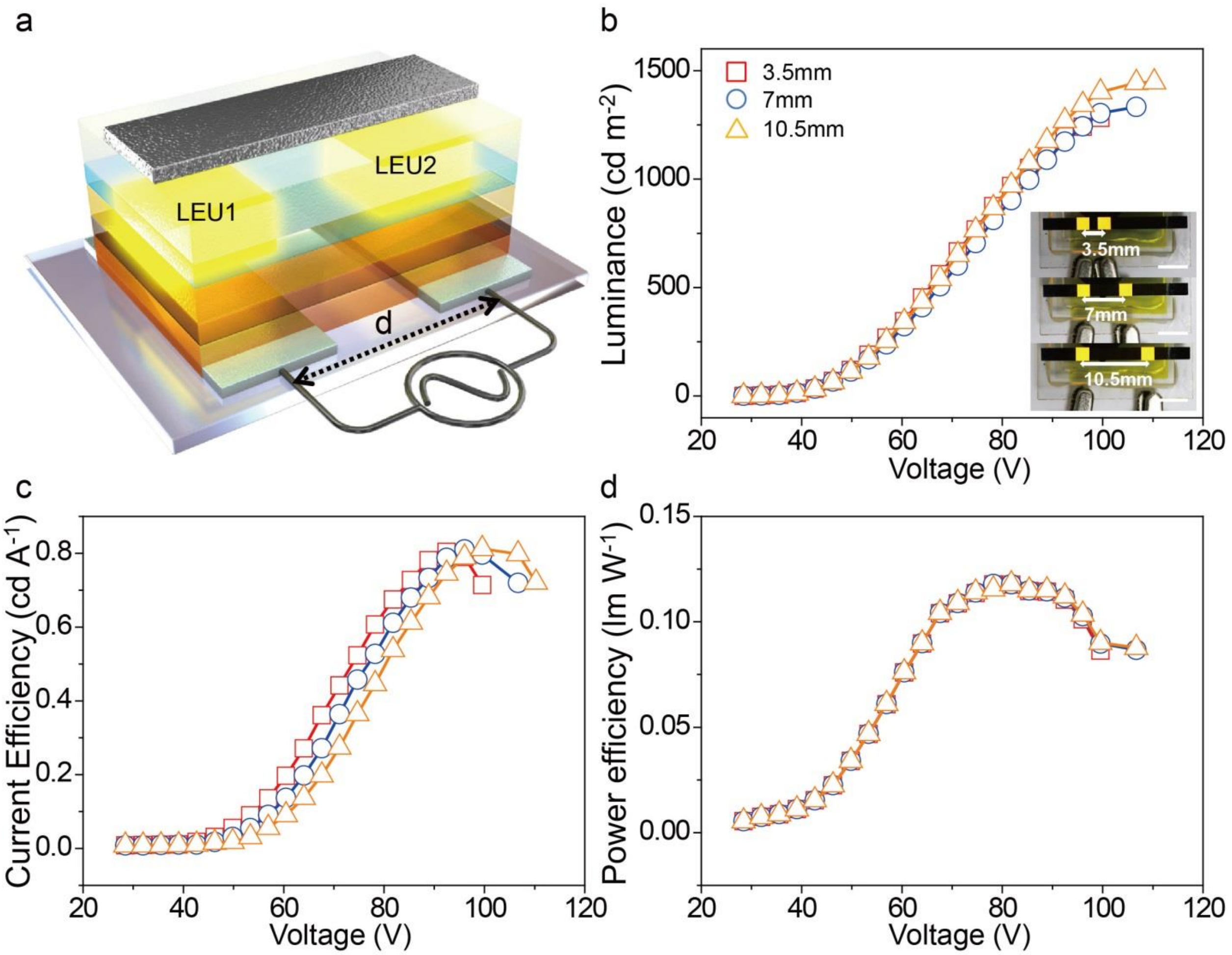

Supplementary Figure 9. Characteristics of parallel AC EL devices as a function of the distance between two parallel bottom electrodes. (**a**) Schematic of a device structure of a parallel AC EL. (**b**) Luminance versus voltage (L–V) characteristics of parallel AC EL devices (**c**), current efficiency (cd A^{-1}) and (**d**) power efficiency (lm W^{-1}) as a function of the distance between two ITO bottom electrodes. The AC frequency was applied of 100 kHz. The photograph in the inset of (b) shows three parallel AC EL devices which turned on with the two bottom electrodes separated by 3.5, 7 and 10.5 mm. The device performance was almost identical, irrespective of the distance between two bottom electrodes. The scale bar is 5 mm.

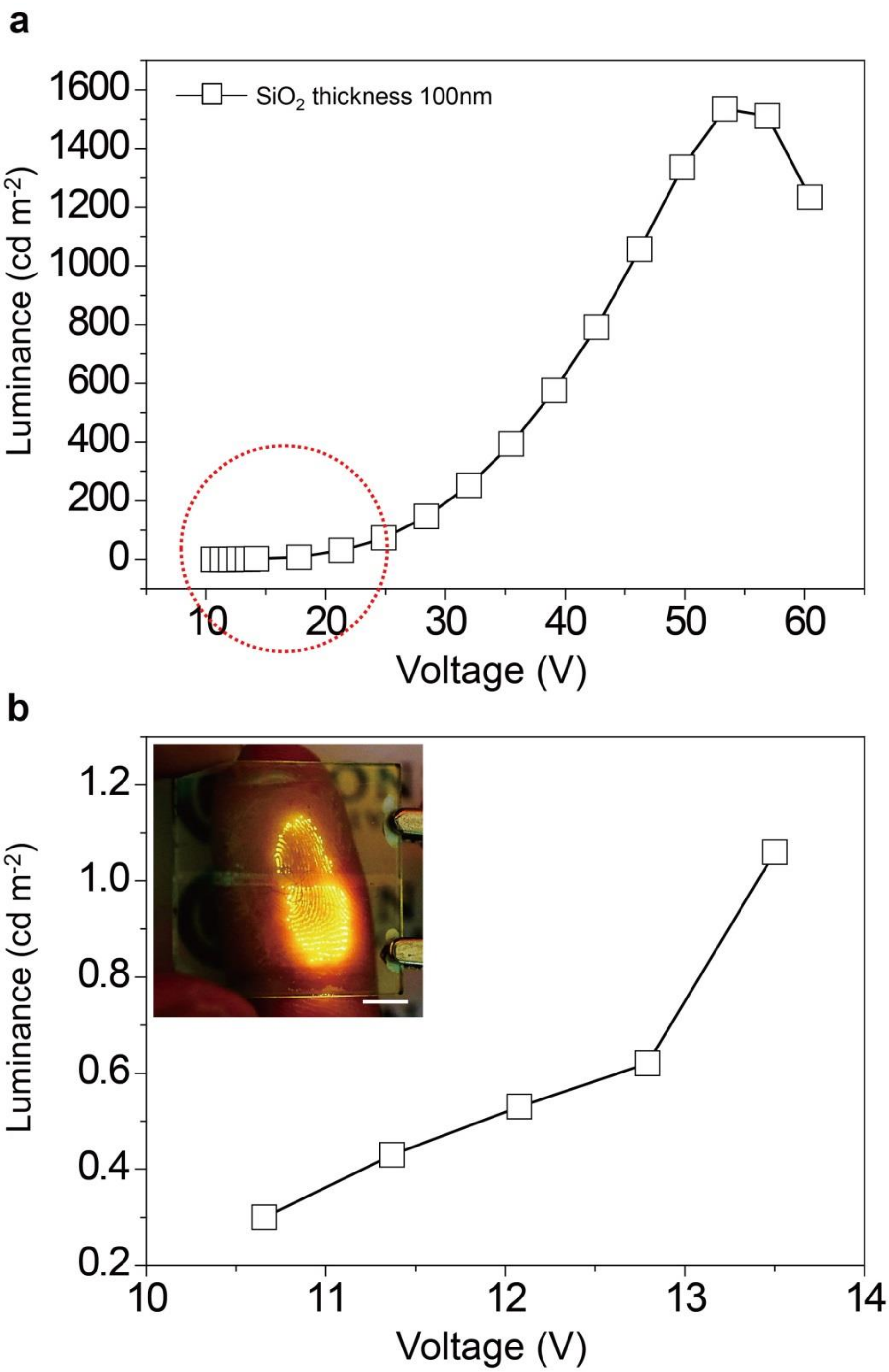

Supplementary Figure 10. A parallel AC EL device with low voltage operation. (**a**)Luminance versus voltage (L–V) characteristics of a parallel AC EL device with ca. 100 nm thick SiO_2 insulator. L-V properties at low voltage regime circled with red dots in (**a**) were zoomed up in (**b**). The photograph in the inset of (b) shows an EL image of fingerprint pattern with 100nm thick SiO_2 insulator at 15 V. The scale bar is 5 mm.

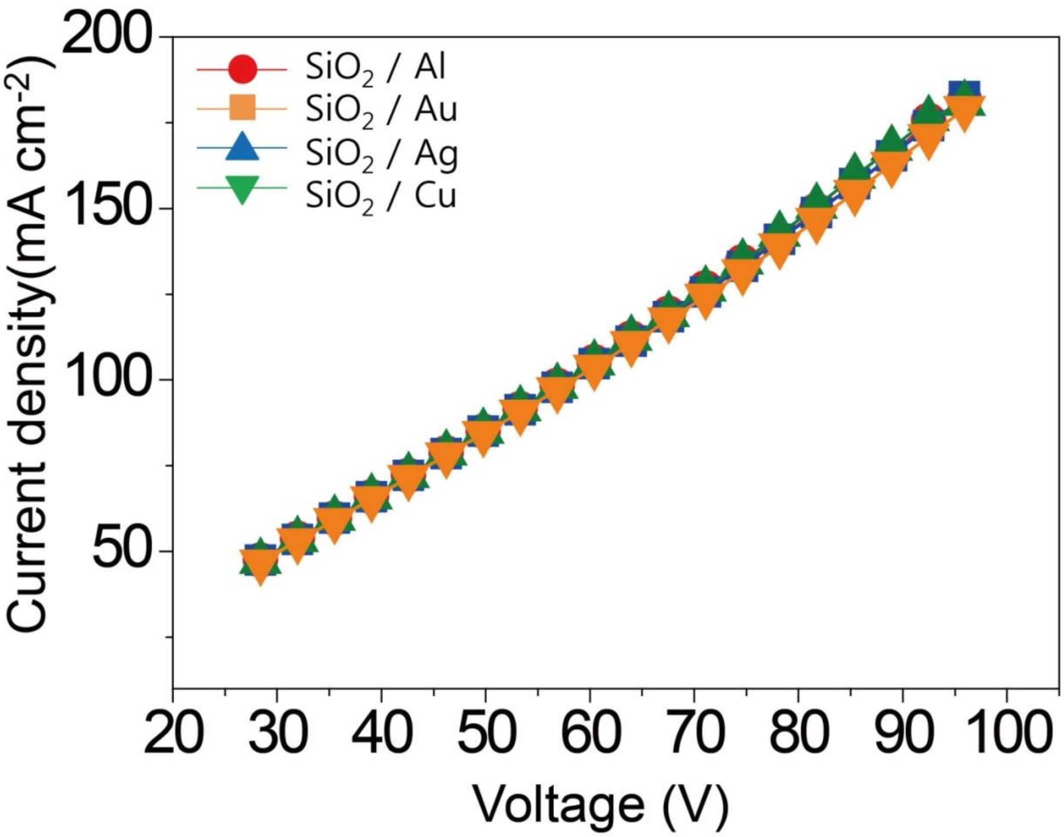

Supplementary Figure 11. Work function independent light emission of a parallel AC EL device. Current density versus voltage characteristics (CE–V) of parallel AC EL devices with four different metal electrodes. The AC frequency was applied of 100 kHz.

Supplementary Note 2:

Light emission vs. conductance of an OLEB

A parallel AC EL device was also examined with a highly conductive PEDOT:PSS film as a top electrode. The highly conductive PEDOT:PSS layer was prepared by adding small amount of DMSO. The device also emitted light but the device performance was, however, worse than that with a conventional metal one due to the lower conductivity of the PEDOT:PSS layer. We also investigated the effect of conductivity of a top electrode on light emission. A series of PEDOT:PSS layers were obtained with different conductivities and sheet resistances by controlling the amount of DMSO in the films as shown in Supplementary Fig. 12a. The sheet resistance value was significantly reduced with DMSO, ranging from approximately 4.5 x 10^5 Ω sq^{-1} to 211 Ω sq^{-1}. While light emission only at edge areas of LEUs was observed in a device with a pristine PEDOT:PSS layer, the rest devices show homogeneous and uniform light emission as shown in Supplementary Fig. 12b. The light emission performance was enhanced with the conductivity of a top electrode. For instance, the maximum luminance values increase with the amount of DMSO, giving rise to approximately 420 cd m^{-2} in a device with a PEDOT:PSS layer containing 5 wt% DMSO (Supplementary Fig. 12c and 12d).

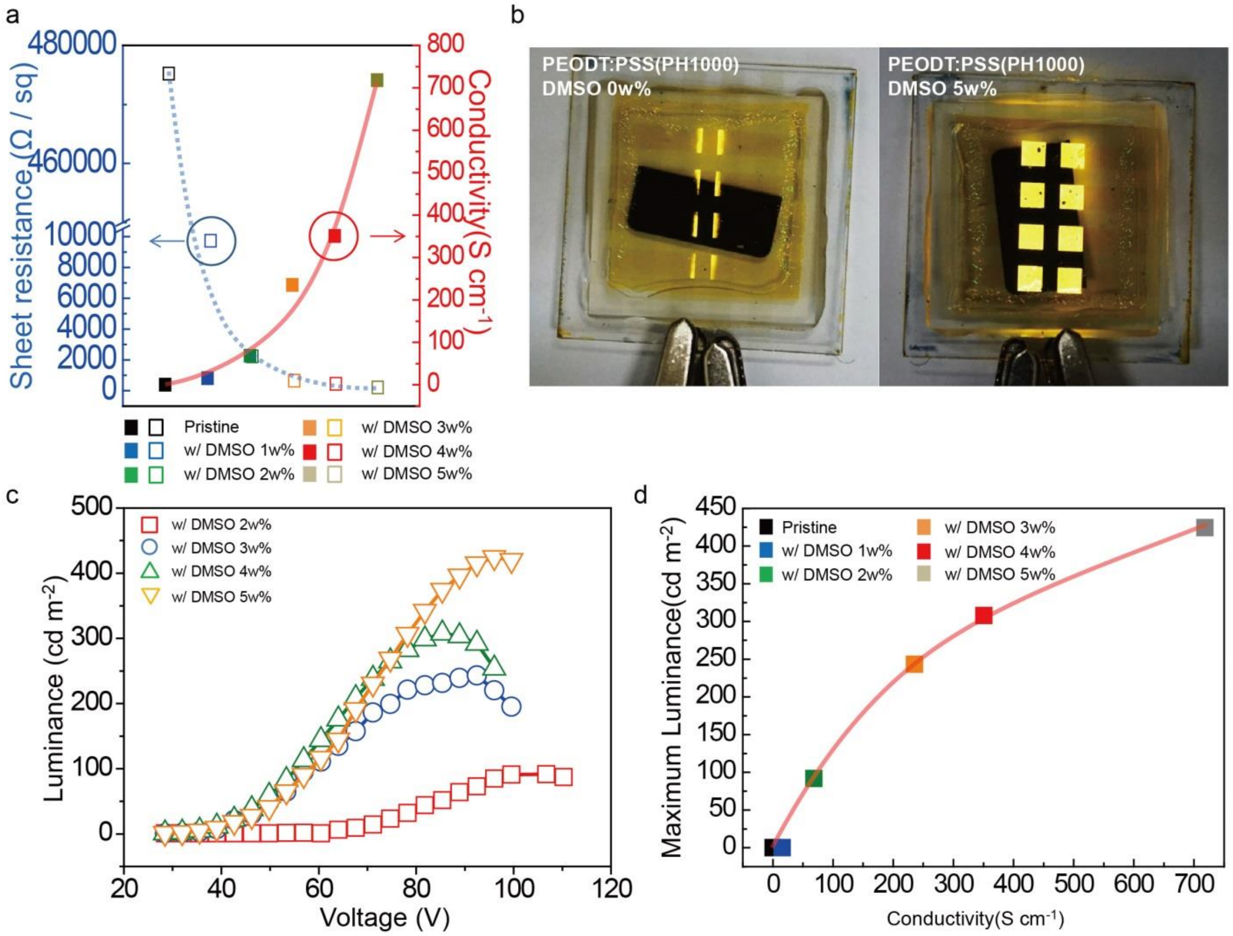

Supplementary Figure 12. The device performance of a parallel AC EL device as a function of conductivity of a top PEDOT:PSS electrode. (**a**) The sheet resistance (open symbols) and conductivity (solid symbols) of PEDOT:PSS electrodes as a function of the amount of DMSO. (**b**) Photographs show light emission of parallel AC EL devices with PEDOT:PSS electrodes containing 0w% (left) and 5wt% DMSO (right). The scale bar is 5 mm. (**c**) Luminance versus voltage (L–V) characteristic of parallel AC EL devices with PEDOT:PSS top electrodes containing different amount of DMSO. (**d**) Maximum luminance characteristics of the devices as a function of the amount of DMSO. The device performance was obtained with the AC frequency of 100 kHz.

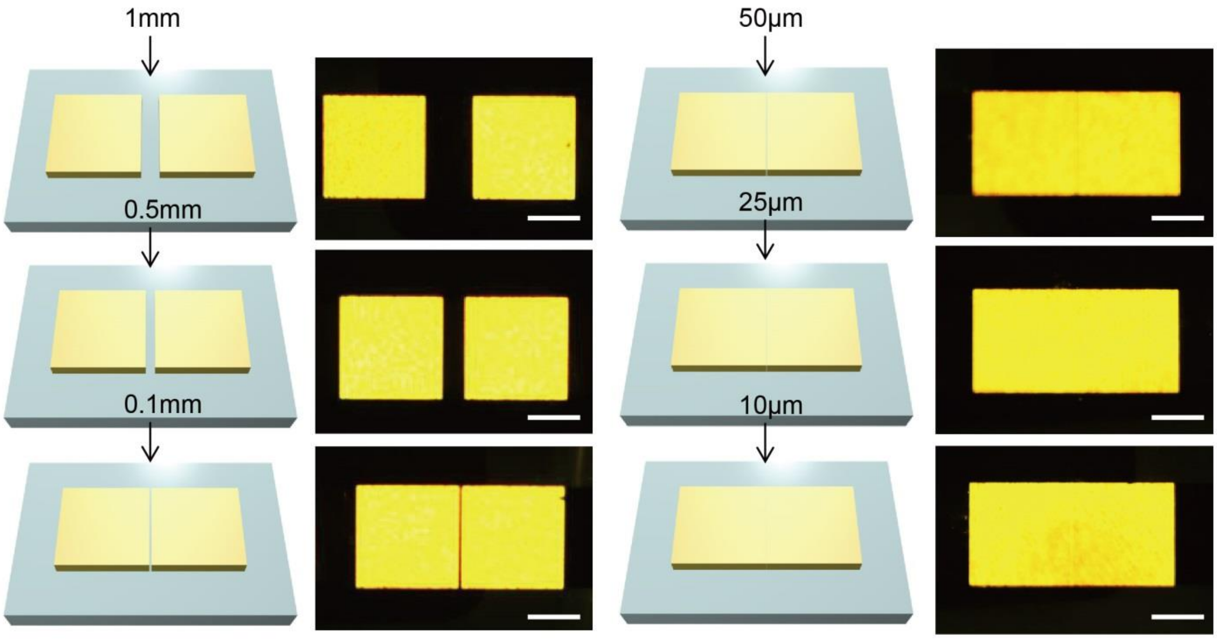

Supplementary Figure 13. Gap dependent luminescence of parallel AC EL devices. Schematics and photographs of parallel AC EL devices with different gaps between two LEUs. The devices were operated at the voltage and the frequency of 30 V and 100 kHz. The gaps are invisible when the gap is 50 μm and below. The scale bar is 1.5 mm

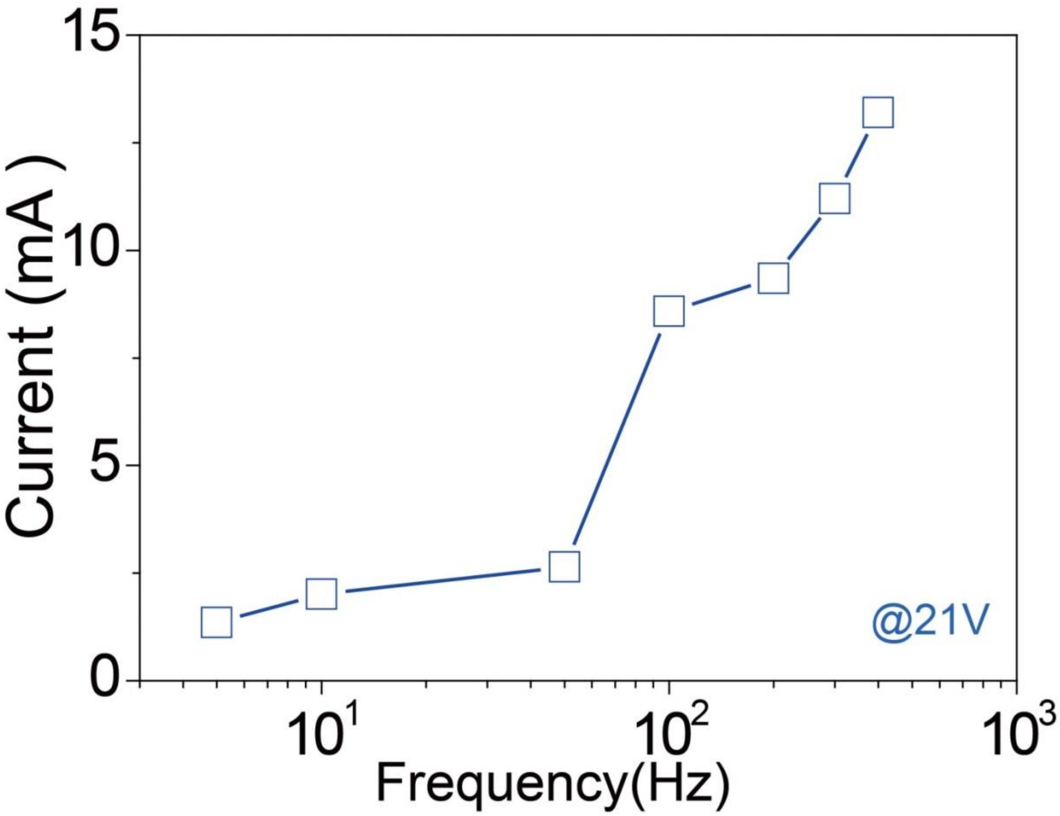

Supplementary Figure 14. Finger detection performance on an OLEB. The variation of device current as a function of frequency under finger contact at 21 V.

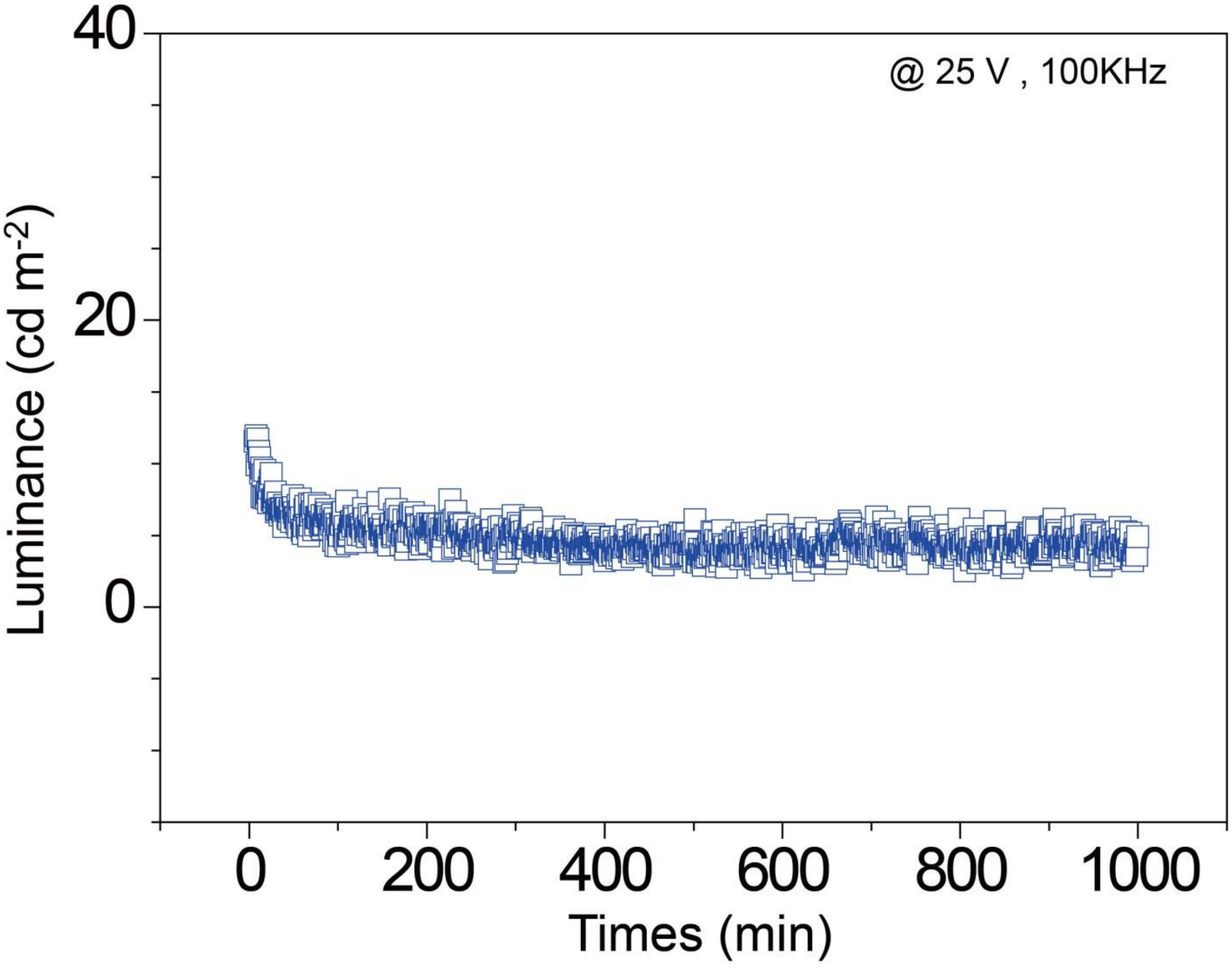

Supplementary Figure 15. Operation stability of a parallel AC EL device. Luminance versus operation time characteristics of a parallel AC EL device. An initial luminescence was 15 cdm^{-2}.

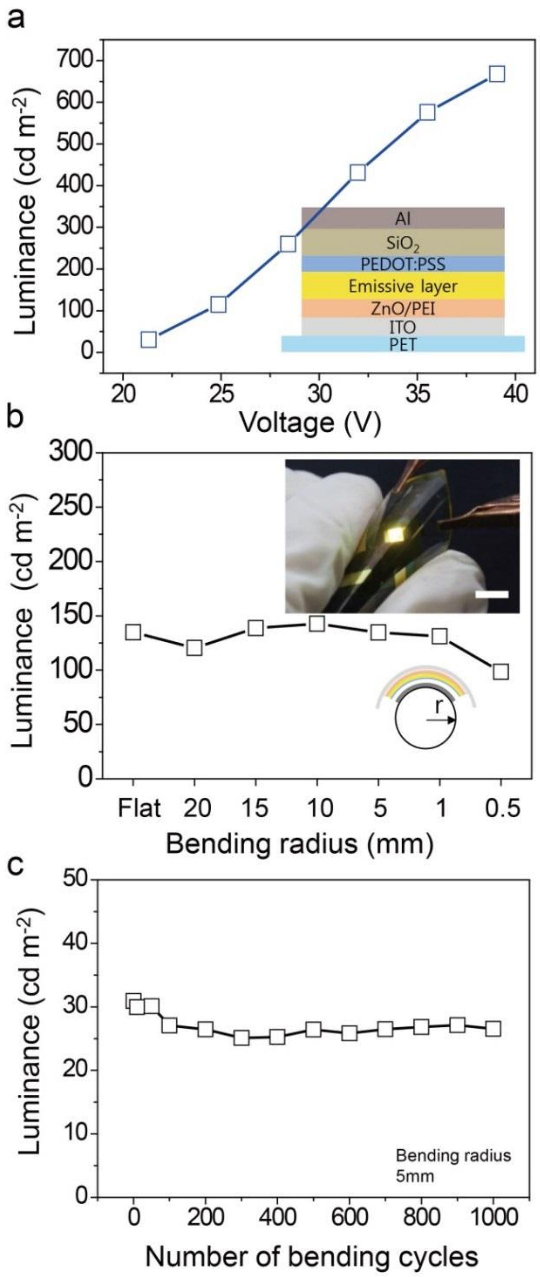

Supplementary Figure 16. Device performance of a flexible OLEB under mechanical deformation. (**a**) Luminance versus voltage characteristics (L–V) of a flexible parallel AC EL device on a PET substrate. (**b**) Luminance variation of a flexible device as a function of bending radius. A photograph in the inset shows the light emission of a device under bending. The device performance was obtained with the AC frequency of 100 kHz. (**c**) Luminance variation as a function of the number of bending cycles. No significant degradation was observed after 1000 cycles. The scale bar is 5 mm.

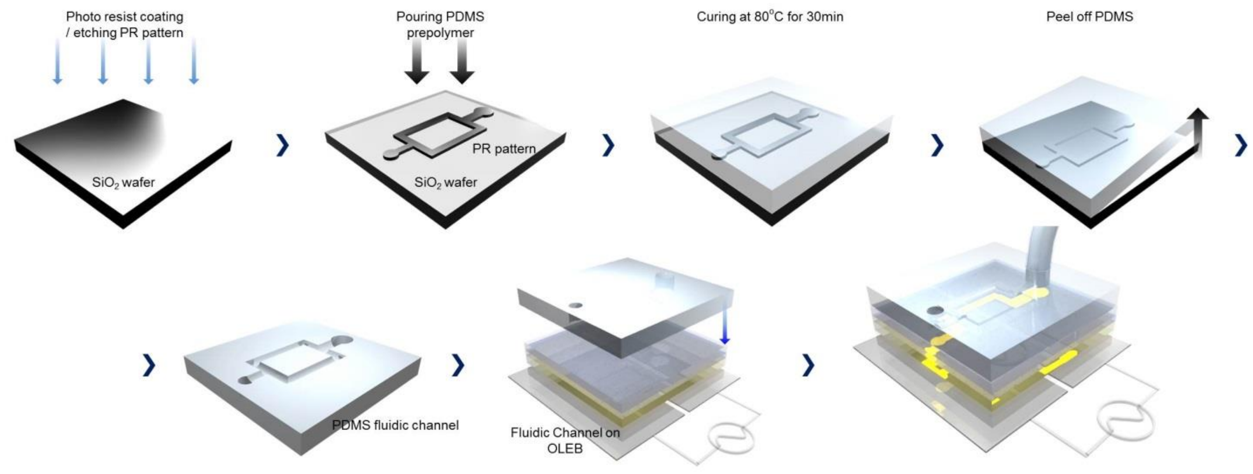

Supplementary Figure 17. The fabrication with fluidic channels on an OLEB. Bifurcated fluidic channel of 500-μm-width for monitoring and visualizing dynamic motion of metallic liquid in the channel was developed by conventional photolithography, followed by channel replication with PDMS. Circular type inlet and outlet were also fabricated.

Sensing Principle	Visualization mechanism	Mechanical property	Device Architecture & components	Operating Voltage (V)	Max. Luminance (cd m^{-2})	Functionality	Ref.
Pressure	Electrochromic color	Stretchable	Electrochromic Device-Pressure Sensor (2)	N/A	N/A	X	3
Pressure	OLED & Electrochromic device	Stretchable & Flexibility	OLED or Electrochromic device-E-skin (2)	4.5	80	X	4
Strain	Thermochromic color	Stretchable	Thermochromic indicator-conductive elastomer (2)	N/A	N/A	X	6
Pressure	Electroluminescence (DC)	Flexible	OLED-Pressure Sensor-TFT (3)	3	3,800	Sensing and mapping	16
Pressure	Electroluminescence (DC)	Stretchable	OLED-Pressure Sensor-Photodetector (3)	9	N/A	X	19
Triboelectric	LED	Flexible	LED-TENG (2)	N/A	N/A	X	20
Triboelectric	Electroluminescence (AC)	Stretchable	Vertical AC-EL (1)	N/A	N/A	Writing	22
Pressure & Strain	Electroluminescence (AC)	Stretchable	Vertical AC-EL (1)	>10,000	N/A	X	17
Pressure & Strain	Electroluminescence (AC)	Stretchable	Vertical AC-EL (1)	>10,000	27.5	Multicolor Display	18
Conductance	Electroluminescence (AC)	Flexible	Parallel AC-EL (1)	20	1,800	Writing, Fingerprint detection & imaging and metallic fluid imaging	Our Work

Supplementary Table 1. The characteristics of user-interactive devices recently reported in the literature and the OLEB present in the current work.
